# The Role of Biologic Therapy Switching to Optimize the Treatment of Severe Pediatric Asthma: Tezepelumab Is a New Therapeutic Alternative for Adolescents

**DOI:** 10.3390/children12081092

**Published:** 2025-08-20

**Authors:** María Erroz Ferrer, Miren Paniagua García, Natividad Viguria Sánchez, Laura Moreno-Galarraga

**Affiliations:** 1 Pediatric Service, University Hospital of Navarra (HUN), 31006 Pamplona, Spainlaura.moreno@unavarra.es (L.M.-G.); 2 IdiSNA, Health Research Institute of Navarra, 31006 Pamplona, Spain; 3 Medicine, Faculty of Health Sciences, Public University of Navarra, (UPNA), 31006 Pamplona, Spain

**Keywords:** asthma, biologics, tezepelumab, omalizumab, pediatric, treatment switch, adherence, cost, personalized medicine

## Abstract

Background/Objectives: The emergence of biologic therapies has transformed the management of severe asthma, offering targeted treatments that improve symptom control and reduce exacerbations. However, taking a biologic solely based on the initial response may not be optimal. Clinicians must regularly reassess the suitability of the selected biologic, considering evolving patient characteristics and the growing availability of treatment options. This article aims to highlight the importance of individualized decision making in the long-term management of pediatric patients receiving biologic therapy, exploring the various reasons that may justify switching biologic treatment. Methods: We present a case to illustrate this approach: a 14-year-old adolescent girl with severe allergic asthma who initially received Omalizumab, achieving clinical, laboratory, and functional improvements. Despite this positive response, the patient reported low-level satisfaction due to the frequency of injections and hospital visits, leading to poor adherence. After a comprehensive reassessment, the treatment was switched to Tezepelumab. Results: Following the switch, the patient maintained stable disease control with a slight improvement in lung function, while adherence, satisfaction, and quality of life improved significantly. The number of hospital visits was reduced from 24 to 12 per year, and the number of injections decreased from 104 to 12 annually. The patient reported high-level satisfaction with the new treatment plan, and the economic burden of therapy was also substantially reduced. We present a table summarizing the essential and secondary factors to consider when initiating or switching biologic therapy. Conclusions: This case highlights the need to go beyond the traditional criteria, such as lack of efficacy and adverse reactions, when evaluating a switch in biologic therapy for pediatric asthma. Factors such as comorbidities, injection frequency and number, route of administration, and cost should also be considered, as they can directly influence adherence and the clinical outcomes in children and adolescents. As the therapeutic options expand, regular reassessment and personalized adjustments remain essential.

## 1. Introduction

Asthma is the most prevalent chronic respiratory condition in children, affecting around 10% of the global pediatric and adolescent population. Severe asthma represents 5–10% of these cases and is associated with a significant symptom burden, morbidity, and mortality [1]. Severe asthma in children and adolescents is associated with poor symptom control, reduced quality of life, and increased healthcare costs, reinforcing the need for therapies that not only improve outcomes, but also enhance cost-effectiveness [2].

Despite notable advances in understanding its pathophysiology over the last decade, asthma attacks and hospitalizations among children remain frequent, and there is still concern about mortality. It is very important in the treatment of severe asthma to first to address modifiable factors, such as treatment adherence, comorbidities, and environmental exposures, but once addressed, some children remain on high-dose therapies, with little control. However, new biologic treatments offer promising options [1].

Omalizumab, a monoclonal antibody directed against IgE, was the first biologic therapy approved in 2003 for the treatment of severe, allergen-induced asthma resistant to standard therapy. Since then, several biologics have been approved for pediatric use, including benralizumab, which is approved by the FDA, but is not currently available in Europe. Each biologic targets specific inflammatory pathways, such as anti-interleukin (IL)-5 (mepolizumab), the IL-5 receptor (benralizumab), the IL-4/IL-13 receptor (dupilumab), and the thymic stromal lymphopoietin (TSLP) (Tezepelumab). The growing range of available biologics with distinct mechanisms of action has introduced new complexities into clinical decision making, including the need to correctly identify the patient’s asthma phenotype or endotype and to establish appropriate criteria for evaluating treatment response and best treatment option [3].

The emergence of all these biologic therapies has clearly transformed the management of severe asthma, offering effective targeted treatments that improve symptom control and reduce exacerbations. However, once a biologic is initiated, it is no longer sufficient to maintain it indefinitely if the response is acceptable. Clinicians must regularly reassess whether the patient is receiving the most appropriate biologic, considering the evolving clinical characteristics, new treatment options, and the expanding indications of these therapies. In the field of pediatrics, biologic therapies are often introduced later than in adult medicine, as pediatric approvals typically follow adult indications once sufficient safety and efficacy data are available. Furthermore, the approved age for administration is frequently lowered gradually as clinical trials extend to younger populations. For this reason, pediatricians must remain well-informed about all the available biologics, understand their specific indications and mechanisms, and stay updated on new developments. Familiarity with the full therapeutic arsenal allows for optimal decision making tailored to each patient [3,4].

In this context, personalized medicine plays a crucial role. The selection of a biologic should not rely solely on traditional markers, such as IgE levels and eosinophil counts, but rather on a comprehensive evaluation of the patient’s phenotype, comorbidities, lifestyle, adherence, and even treatment burden, treatment posology, and treatment cost.

Tezepelumab, a monoclonal antibody that blocks thymic stromal lymphopoietin (TSLP), has recently emerged as a broad-acting agent that targets upstream inflammatory pathways, regardless of the allergic or eosinophilic phenotype [5,6,7,8]. It is currently available for patients aged 12 years and older with uncontrolled severe asthma, making it a viable option in pediatric care. Nopsopon T. et al. published their study comparing the efficacy of Tezepelumab with dupilumab, benralizumab, and mepolizumab in eosinophilic asthma, performing a systematic review and Bayesian network meta-analysis of 10 randomized controlled trials (n = 9201). This meta-analysis referred mainly to adult studies (mean age 49.6 years), although some of them included children aged ≥12 years. Tezepelumab was linked to significantly lower exacerbation rates than benralizumab. Tezepelumab, mepolizumab, and dupilumab all had >99% probability of reducing exacerbations versus a placebo, and also tezepelumab and dupilumab improved FEV1 by ≥100 mL versus placebo [9]. Overall, Tezepelumab and dupilumab showed greater improvements in exacerbations and lung function than benralizumab or mepolizumab, though below clinical significance thresholds.

The PASSAGE study evaluated the real-world effectiveness and safety of Tezepelumab in 208 patients with uncontrolled severe asthma, including adolescents. With a regimen of 210 mg every 4 weeks over a 52-week period, the treatment resulted in a 76% reduction in the annual asthma exacerbation rate. Significant improvements were observed in lung function (overall increase of 0.11 L in pre-bronchodilator FEV1), symptom control (ACQ-6 score reduction of −1.1), and quality of life. Inflammatory biomarker values, such as eosinophil counts and IgE levels, were also significantly reduced. In terms of safety, only 7.7% of patients experienced serious adverse events, and the discontinuation rate due to adverse events was minimal (0.5%) [10].

Publishing real-life clinical experiences is essential to complement the efficacy data from randomized controlled trials. As switching and even combining biologics becomes increasingly common in clinical practice, real-world data are crucial for understanding treatment effectiveness, safety, and outcomes beyond controlled settings and may help guide future research and therapeutic strategies. This report discusses the rationale for switching biologic therapy in adolescents with severe asthma, beyond the traditional criteria, and demonstrates it with a recent, real-life, clinical experience.

## 2. Clinical Context and Biologic Switch

Switching a medication, including biologic therapy, may be considered for several reasons. Traditionally, changes are prompted by an insufficient clinical response, the emergence of adverse effects, or newly identified contraindications. However, in current clinical practice—particularly in pediatric asthma—other important factors must also be taken into account. These include poor adherence due to complex dosing schedules or injection frequency, treatment burden on the patient and family, cost considerations, and the availability of new agents with improved mechanisms of action or broader indications. Additionally, changes in the patient’s clinical phenotype, comorbidities, or personal preferences may also support the decision to modify treatment to achieve the most effective and sustainable long-term outcomes.

We present a case from our Severe Asthma Clinic (SAC) as an example in which a switch from Omalizumab to Tezepelumab was performed, despite the patient being well controlled with Omalizumab, experiencing no adverse effects, and showing improved lung function and good clinical control.

A 14-year-old Black adolescent girl (69 kg) with a diagnosis of severe allergic asthma and a type two inflammatory phenotype presented with poor asthma control despite a four-step treatment, which consisted of high-dose Inhaled Corticosteroids (ICSs) combined with a Long-Acting Beta-Agonist (LABA) and montelukast. Her adherence to the treatment was good, and her inhalation technique had been verified as correct. Lung function was severely impaired, with a Forced Expiratory Volume in one second (FEV1) of 48%, a Forced Vital Capacity (FVC) of 65%, and an FEV1/FVC ratio of 64% (spirometry values are expressed as percentages of predicted values according to the Global Lung Function Initiative (GLI) 2012 reference equations for pediatric patients). Fractional exhaled nitric oxide (FeNO) was elevated (>50 ppb), and total serum IgE was markedly high (1300 UI/mL), though the peripheral blood eosinophil count remained low (100/µL). She was allergic to dust mites and cockroaches, with documented ongoing exposure to both allergens.

In July 2021, subcutaneous Omalizumab therapy was initiated at a dose of 600 mg every two weeks, calculated according to her weight and IgE levels. The patient was monitored in the SAC, showing clear clinical and spirometric improvements (FEV1 73%, FVC 85%, and FEV1/FVC 76%), with no asthma exacerbations, no emergency visits or hospital admissions, no need for oral corticosteroids, and no adverse effects related to the medication in the year after Omalizumab initiation. She had a complex family situation, which the care team also attempted to address and support. Although asthma control remained good and the maintenance treatment was successfully reduced, over time the patient began to miss follow-up appointments and presented with increasing distress. She reported intense fear of the injections, often arriving at the hospital in tears. Due to her severe needle phobia and the unstable home environment, she was not considered a candidate for home administration of the treatment, which meant she had to attend the hospital every two weeks to receive her Omalizumab doses. This increasingly frequent burden contributed to a progressive decline in adherence, despite the good clinical response.

Given the progressive emotional distress caused by the frequent hospital visits and injections, as well as the growing impact on both the patient and her family, the SAC team considered a therapeutic alternative. In 2024, a switch to Tezepelumab was initiated, with a fixed monthly subcutaneous dose of 210 mg, regardless of her weight or IgE levels. This biologic offered a simpler and less-burdensome treatment regimen, better suited to the patient’s needs.

Following the switch, the patient maintained good clinical control and demonstrated even further improvements in pulmonary function (FEV1 97%, FVC 114%, and FEV1/FVC 77%). Her FeNO levels normalized, and she remained free of exacerbations or hospital admissions. Her IgE levels before starting biologic therapy were 1300 UI/mL. After treatment with Omalizumab, they decreased markedly to 274 UI/mL. Following the switch to Tezepelumab, her IgE levels were 656 UI/mL in January 2024, and increased slightly to 731 UI/mL by January 2025, remaining well below the pre-treatment baseline values. The number of hospital visits dropped from 24 to 12 per year, and the number of injections was reduced from 104 to just 12 annually. The patient and her family reported a high level of satisfaction with the new treatment plan, and the economic burden was also reduced, with the annual cost of therapy decreasing from EUR 30,000 to EUR 8000.

Here is a table that describes the evolution of asthma control, pulmonary function and treatment burden before the biologic, after Omalizumab, and after the biologic switch to Tezepelumab (Table 1).

## 3. Discussion

Globally, poor adherence remains a significant barrier to effective healthcare delivery. According to the World Health Organization, only around 50% of patients with chronic conditions follow prescribed treatment regimens, with especially poor adherence observed in asthma, diabetes, and hypertension therapies [11].

One of the most critical barriers to treatment adherence is the complexity of therapeutic regimens, which goes beyond the number of medications and also includes other factors, such as the route of administration, frequency, and the burden associated with each treatment. Additional challenges include limited health literacy, poor understanding of the benefits of therapy, side effects, dissatisfaction with treatment, high medication costs, and weak communication between patients and healthcare providers. While various strategies have been implemented—such as simplifying regimens—their effectiveness varies widely [12].

In pediatric and adolescent populations, fear of needles and rejection of frequent injections are common and relevant barriers to treatment adherence. All biologic therapies currently approved for use in children and adolescents are administered subcutaneously. Furthermore, in certain cases—particularly those requiring weight- and IgE-based dosing, such as Omalizumab—the treatment regimen may involve up to four injections every two weeks, significantly increasing the treatment burden for the patient and the family.

In the case presented as an example, although the patient was clinically stable, the decision was made in the SAC to switch to Tezepelumab (210 mg SC every four weeks). This change resulted in additional improvement in lung function, but more importantly, it enhanced adherence and reduced the emotional and logistical burden on the family, and the patient. The number of clinic visits to the SAC dropped from 24 to 12 per year, injections from 104 to 12, and the annual treatment cost from approximately EUR 30,000 to EUR 8000. By January 2025, spirometry showed even further improvement (FVC 114%, FEV1 97%, and FEV1/FVC 77%), FeNO normalized, IgE increased slightly to 731 UI/mL (below pre-treatment baseline values), and no exacerbations or adverse events were reported. The patient and her family reported a marked improvement with the new regimen, and the level of adherence to scheduled visits reached 100%, with a high level of satisfaction.

In some cases, as the one presented, the biologic switch from Omalizumab to Tezepelumab grants asthma control and lung function, while being convenient, improving adherence, and being cost-effective. These real-life outcomes are consistent with clinical trials showing Tezepelumab’s efficacy across different phenotypes, including patients with low eosinophil counts or non-allergic asthma [5,6,7,8].

Until recently, Omalizumab was the only biologic approved for pediatric severe asthma. Today, the pediatric therapeutic landscape has expanded to include Mepolizumab, Dupilumab, and Tezepelumab, with Benralizumab also available in some countries [4,5,6,7,8,9,10]. This requires clinicians to go beyond the classical criteria when considering a biologic switch. Individualized decision making should integrate factors such as patient phenotype, comorbidities, injection frequency, adherence, and economic cost.

Biologic therapy in pediatric asthma should be dynamic, with regular reassessment of its suitability for each patient. The goal is not only to achieve clinical control, but to ensure that the chosen treatment remains the most appropriate option as the patient grows, circumstances evolve, and new therapies become available. In the current context, where multiple biologics are approved for pediatric use, decisions about switching should go beyond the traditional criteria, such as lack of efficacy and adverse effects. Other practical aspects—including dosing frequency, number of injections, impact on daily life, and affordability—play a crucial role in adherence and overall patient and family satisfaction. These factors are especially relevant in children and adolescents, where treatment success often depends on minimizing discomfort and maximizing autonomy.

As summarized in Table 2, the first selection or a switch in biologic therapy for severe asthma should be based on a combination of essential and secondary factors. Essential factors—such as the patient phenotype, comorbidities, age, and later clinical response—must be assessed both before initiating treatment and during follow-up. In parallel, secondary factors, like the route and frequency of administration, the number of injections, possibility of home administration, patient preferences, and treatment cost, also play a crucial role in adherence and overall satisfaction and should not be overlooked in clinical decision making. It is important to maintain openness to change. While we are used to switching medications only when they are ineffective [13], the increasing availability of new treatment options challenges us to reconsider this approach. We may now encounter situations where it is appropriate to switch therapies that are working well if there is evidence that another option could provide even better outcomes.

## 4. Conclusions

In conclusion, biologic selection for pediatric asthma should be part of a personalized, patient-centered approach that balances clinical efficacy with real-life applicability, ensuring that the right biologic is chosen for the right patient at the right time [13,14].

Decisions to switch biologic therapy should be based not only on the traditional indicators, but also on the practical considerations—including comorbidities, route and frequency of administration, number of injections, treatment cost, and patient and family preferences—which can have a direct impact on adherence, satisfaction, and long-term outcomes.

Tezepelumab offers a valuable and versatile alternative for patients with severe asthma, particularly those with non-allergic phenotypes, adherence challenges, or a preference for a simpler regimen. Its broad mechanism of action and convenient monthly dosing, administered as a single injection, make it an attractive option across a wide range of clinical scenarios.

## Figures and Tables

**Table 1:** Evolution of asthma control, pulmonary function, and treatment burden before biologic, after Omalizumab, and after biologic switch to Tezepelumab.

	Pre-Biologic	Omalizumab Initiation	Tezepelumab Switch
**Start date**		July 2021	January 2024
**Dose**		600 mg	210 mg
**Number of injections/year**		104	12
**Injection frequency**		Every 2 weeks (4 injections/dose)	Every 4 weeks (1 injection/dose)
**Clinical control**	Not treated in SAC >6 ER visits, 1 hospitalization, and >3 courses of oral corticosteroids in previous year	24 visits SAC/year No ER visits, no hospitalizations, and no courses of oral corticosteroids	12 visits SAC/year No ER visits, no hospitalizations, and no courses of oral corticosteroids
**Basal treatment**	High-dose ICS-LABA + Montelukast	Medium-dose ICS-LABA	Medium-dose ICS-LABA
**Pulmonary function**	FEV1 48%, FVC 65%, FEV1/FVC 64% Not very obstructive	FEV1 73%, FVC 85%, FEV1/FVC 76% Somewhat obstructive	FEV1 97%, FVC 114%, FEV1/FVC 77% Normal
**FeNO levels (ppb)**	51 ppb	25 ppb	16 ppb
**IgE levels (UI/mL)**	1300	274	656
**Blood Eosinophils**	100	100	100
**Asthma control**	Poor	Good	Good
**ACT score**	12	25	25
**Adverse effects**	—	None	None
**Patient satisfaction**	Very Low	Low	High
**Family satisfaction**	Very Low	Improved	High

Abbreviations used in this table: SAC: Severe Asthma Clinic; ER: Emergency Room; FeNO: Fraction of Exhaled Nitric Oxide; ICS: Inhaled Corticosteroid; LABA: Long-Acting Beta-Agonist; FEV1: Forced Expiratory Volume in one second; FVC: Forced Vital Capacity; ppb: parts per billion; ACT: Asthma Control Test.

**Table 2:** Key clinical and practical factors to consider when initiating or switching biologic therapy in severe asthma.

	Before Starting Biologic	After Starting Biologic
**Essential Factors**	Patient phenotype	Clinical control/ACT
	Patient age	Changes in Lung function
	Comorbidities	Changes in biomarkers (e.g., FeNO, IgE, eosinophils)
	Availability of Biologic (local indications)	Adverse Events
**Secondary Factors**	Route of administration (iv/im/sc/po)	Patient and family satisfaction
	Injection frequency if enteral (Number of injections/year)	Family/caregiver burden
		Adherence
	Home vs. hospital-administration	
	Patient and family preferences	
	Treatment cost	

Abbreviations used in thus table: FeNO: Fraction of Exhaled Nitric Oxide; IgE: immunoglobulin E; iv: intravenous; im: intramuscular; sc: subcutaneous; po: per oral.

## Data Availability

No new data were created or analyzed in this study. Data sharing is not applicable to this article.

## Abbreviations

The following abbreviations are used in this manuscript:

TSLP	Thymic Stromal Lymphopoietin
IgE	Immunoglobulin E
ACQ-6	Asthma Control Questionnarie-6
SAC	Severe Asthma Clinic
ICS	Inhaled Corticosteroid
FEV1	Forced Expiratory Volume in one second
FVC	Forced Vital Capacity
FeNO	Fraction of Exhaled Nitric Oxide
Ppb	Parts per billion
ER	Emergency Room
LABA	Long-Acting Beta-Agonist
ACT	Asthma Control Test
iv	Intravenous
im	Intramuscular
sc	Subcutaneous
po	Oral
